# Reconfigurable electronics by disassembling and reassembling van der Waals heterostructures

**DOI:** 10.1038/s41467-021-22118-y

**Published:** 2021-03-23

**Authors:** Quanyang Tao, Ruixia Wu, Qianyuan Li, Lingan Kong, Yang Chen, Jiayang Jiang, Zheyi Lu, Bailing Li, Wanying Li, Zhiwei Li, Liting Liu, Xidong Duan, Lei Liao, Yuan Liu

**Affiliations:** 1 Key Laboratory for Micro-Nano Optoelectronic Devices of Ministry of Education, School of Physics and Electronics, Hunan University, Changsha, China; 2 State Key Laboratory for Chemo/Biosensing and Chemometrics, College of Chemistry and Chemical Engineering, Hunan University, Changsha, China

**Keywords:** Electrical and electronic engineering, Electronic devices, Two-dimensional materials

## Abstract

Van der Waals heterostructures (vdWHs) have attracted tremendous interest owing to the ability to assemble diverse building blocks without the constraints of lattice matching and processing compatibility. However, once assembled, the fabricated vdWHs can hardly be separated into individual building blocks for further manipulation, mainly due to technical difficulties in the disassembling process. Here, we show a method to disassemble the as-fabricated vdWHs into individual building blocks, which can be further reassembled into new vdWHs with different device functionalities. With this technique, we demonstrate reconfigurable transistors from n-type to p-type and back-gate to dual-gate structures through re-stacking. Furthermore, reconfigurable device behaviors from floating gate memory to Schottky diode and reconfigurable anisotropic Raman behaviors have been obtained through layer re-sequencing and re-twisting, respectively. Our results could lead to a reverse engineering concept of disassembled vdWHs electronics in parallel with state-of-the-art vdWHs electronics, offering a general method for multi-functional pluggable electronics and optoelectronics with limited material building blocks.

## Abstract (web summary)

Here, the authors report a method to fabricate reconfigurable electronic devices based on 2D materials by using polyvinyl alcohol as substrate. This technique enables repeatable disassembling and reassembling of van der Waals heterostructures with different functionalities.

## Introduction

With dangling-bond-free surface, two-dimensional (2D) layered materials can be isolated and combined to create a wide range of van der Waals heterostructures (vdWHs) without the constraints of conventional lattice matching and processing compatibility^1–12^. With a decade of intensive efforts, vdWHs have become an independent research field, offering a rich playground for both fundamental studies^13–17^ as well as novel device concepts^18,19^. On the other hand, with weak bonding forces towards neighbor layers, the artificial stacked vdWHs could theoretically be disassembled into individual 2D building blocks^20^ and stacked again with different combinations, sequences, angles and hence different device functions, similar as building diverse structures with same pieces of toy bricks^3^. However, this possibility has not been demonstrated and could be largely attributed to technical difficulties in the disassembling process, greatly limiting vdWHs for high-performance devices as well as for investigating fundamental physics. For example, twisting two graphene monolayers into magic angle (1.1°) have enabled unique correlated insulator behavior and superconductivity^21,22^, and similarly, twisting two monolayers of transition metal dichalcogenides have demonstrated tunable moiré potentials inside hetero-bilayers with designable optical and electronic states^23–26^. However, the rotation angle of conventional vdWHs can not be changed once assembled. To identify the accurate angle, current approaches rely on fabricating and measuring large amounts of samples with different twist angles, which is not only a tedious process but also limited by the difficulties to identify the exact stacking angle. More importantly, the material quality (e.g., defects, dopants) and interface condition (e.g., surface contaminations) could vary a lot across different samples, posing key technique challenges for accurately investigating the fundamental physics. Although recent report demonstrates in situ twisting of the vdWH using an atomic force microscopy (AFM) tip as the nano-manipulator^27^, it requires complex setup and the 2D material need to be etched into a designed structure with pushing arm, hindering its practical application as well as fundamental device measurement.

Here, we report a simple approach to fully disassemble the fabricated vdWHs into individual building blocks, by utilizing atomically flat polymers as the handling substrate. The disassembled individual layers can be further reassembled into new vdWHs with distinct material combinations, sequences, and twisting angles, and hence diverse device functions. With this technique, we demonstrate the same MoS_2_ flake can be reconfigured from n-type to p-type transistors or from back-gate into top-gate device structures, by re-stacking different contact metals and dielectrics. Furthermore, this method could be extended to multi-layer vdWHs, where a four-layer (graphene/BN/MoS_2_/metal) non-volatile memory is successfully disassembled and re-stacked with a different sequence, and the device function is reconfigured into a Schottky diode. These highly reconfigurable devices are intrinsically different from conventional electronic devices, in which the essential building blocks (e.g., contact, dielectric or barrier layer) are strongly bonded (through covalent or ionic bonds) once deposited and device functions are fixed once fabricated. Finally, we demonstrate a vdWH can be disassembled and re-twisted multiple times of any designed angles with twisting resolution <0.1°, leading to the observation of highly switchable anisotropic behaviors. Our results not only demonstrate the possibility of re-stacking diverse material building blocks at atomic level with reconfigurable functions, but also offer a general method to manipulate twistable electronics with ultra-high resolution while ensuring identical materials quality. These proof-of-concept devices could also intrigue a reverse engineering concept of disassembled vdWHs electronics or reassembled vdWHs electronics in parallel of state-of-the-art vdWHs electronics, providing rich implications for multi-functional pluggable electronics, optoelectronics with limited material building blocks.

## Results

### Disassembling and reassembling processes of bilayer vdWH

**Fig. 1:**
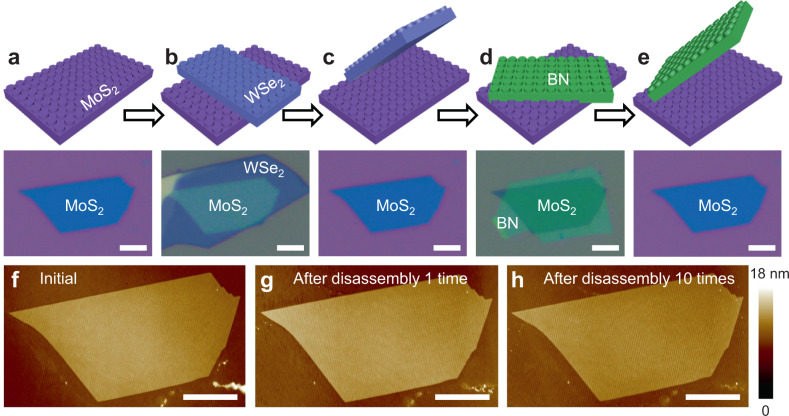
Characterization of the disassembling/reassembling processes of a bilayer vdWH. **a**–**e** Schematics and the optical images of the disassembling/reassembling processes: MoS_2_ exfoliation on PVA substrate (**a**), WSe_2_ dry transferred on top of MoS_2_ (**b**), WSe_2_ physically disassembled from MoS_2_ surface (**c**), BN dry transferred onto the pre-disassembled MoS_2_ (**d**), and BN disassembled from MoS_2_ after 10 times (**e**). **f** AFM image of the as-exfoliated MoS_2_ with pristine surface. **g**, **h** AFM images of the MoS_2_ surface after 1 time (**g**) and 10 times (**h**) disassembling/reassembling processes. The clean and pristine MoS_2_ surface observed here is the result of dry disassembling process, and is essential for further vdW integration. Scale bars, 5 μm.

Figure 1 shows the standard disassembling and reassembling processes, using a bilayer vdWH as an example. First, a few-layer MoS_2_ flake (~4 nm thick) is mechanically exfoliated on top of atomically flat polyvinyl alcohol (PVA) substrate (Fig. 1a). Next, another WSe_2_ flake (~5 nm thick) is dry transferred on top of MoS_2_ using PVA handling substrate, resulting in a typical MoS_2_/WSe_2_ vdWH, as shown in Fig. 1b. Here, the atomically flat PVA substrate could ensure the intimate contact and strong interaction between substrates and 2D materials^28,29^ (compared to the weak vdW force within MoS_2_/WSe_2_ heterostructure), which is essential to avoid the MoS_2_/substrate separation during following disassembling process. This is in great contrast to conventional transfer techniques using polydimethylsiloxane (PDMS)^30^ or polypropylene carbonate (PPC)^31^ as the handling polymers, where the whole vdWH stack would be either left on the substrate or picked up by the polymer, as schematically illustrated in Supplementary Fig. 1. The detailed fabrication process of polymer with atomically flat surface is shown in Methods and Supplementary Fig. 2. Once forming vdWH, the top WSe_2_ can be separated from the bottom MoS_2_ by mechanically removing its PVA handling polymer substrate (Fig. 1c), due to the weaker vdW bonding between MoS_2_ and WSe_2_ (compared to stronger bonding within MoS_2_ and PVA substrate). After the disassembling process, the MoS_2_ still demonstrates flat surface similar to pristine MoS_2_, as shown AFM measurement in Fig. 1f, g. This observation is resulted from the dry disassembling process used here, without involving any wet chemicals, surface contaminations, or polymer residues, mimicking a reverse engineering of the dry-transfer process. With pristine surface, the disassembled MoS_2_ can be further used as an individual building block to form new vdWHs. As shown in Fig. 1d, e, another layer of BN could be vdW integrated on the same MoS_2_ flake to form BN/MoS_2_ heterostructure, and then mechanically disassembled again using previous described method. The disassembling and reassembling processes are highly reliable, where the MoS_2_ still demonstrates fresh surface after ten repetitive cycles, as shown in the AFM image in Fig. 1h. We note the method is not only limited to bilayer vdWHs, but could be well extended to multi-layer devices or more complicated structures by involving state-of-the-art pickup transfer technique, and will be discussed in detail.

### Reconfigurable MoS_2_ devices with different contact metals

**Fig. 2:**
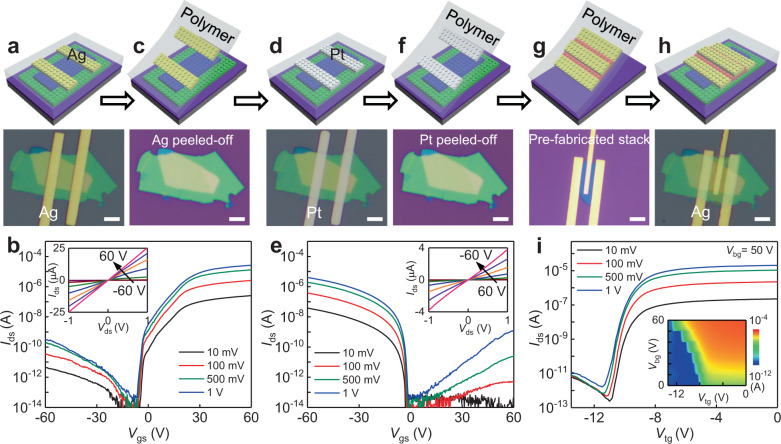
Reconfigurable transistors by vdW disassembling and integrating various contact metals and dielectric on the same MoS_2_ channel. **a** Schematic and the optical image of Ag electrode pairs vdW laminated onto a MoS_2_ channel. **b** *I*_ds_–*V*_gs_ transfer characteristics and *I*_ds_–*V*_ds_ (inset) output curve of the MoS_2_ transistor with vdW Ag electrodes, demonstrating n-type device characteristic with ohmic contact. **c** Schematic and the optical image of Ag electrode pairs physically disassembled from MoS_2_ surface. The disassembled MoS_2_ demonstrates clean surface that is essential for further integration. **d** Schematic and the optical image of Pt electrode pairs vdW laminated on the pre-disassembled MoS_2_ channel. **e** *I*_ds_–*V*_gs_ transfer characteristics and *I*_ds_–*V*_ds_ (inset) output curve of the MoS_2_ transistor with vdW Pt electrodes, demonstrating the switch of device polarity (from n-type to p-type) simply using different contact metals, due to energy match between high work function Pt and the valance band of MoS_2_. **f** Schematic and the optical image of Pt electrode pairs physically disassembled again from MoS_2_ surface. **g**, **h** Top-gate stack (consisting BN dielectric and Ag gate metal) and contact electrode pairs (Ag) are pre-fabricated (**g**), and vdW laminated onto pre-disassembled MoS_2_ channel (**h**), leading to the fabrication of dual-gate device with same MoS_2_ channel. **i** The device demonstrates n-type behavior with dual-gate modulation, and the inset shows two-dimensional plot of the *I*_ds_ as a function of *V*_tg_ and *V*_bg_. Scale bars are 5 μm in optical images.

The ability to disassemble the fabricated vdWHs into pristine individual layers, could enable unique reconfigurable transistors by hot-plugging different vdW metals as contact electrode and insulating materials as gate dielectric. For example, using a MoS_2_ flake as the semiconductor channel and BN/SiO_2_ (60 nm/300 nm thick) as the back-gate dielectric, typical n-type transistor behavior is observed by vdW assembling Ag metal pairs as the electrodes^32^ (Fig. 2a). As shown in Fig. 2b, the assembled transistor demonstrates n-type *I*_ds_–*V*_gs_ transfer curve with on-off ratio over 10^9^, as well as linear *I*_ds_–*V*_ds_ output characteristics (Fig. 2b, inset), suggesting the ohmic contact between low work function Ag and underlying MoS_2_, consistent with previous reports^33–35^. In particular, the threshold voltage of the n-type transistor is close to 0 (Fig. 2b), indicating the optimized interface during vdW integration without additional doping effect. Next, the Ag electrode pairs are mechanically separated from MoS_2_ channel using the disassembling technique (Fig. 2c), where another pair of pre-fabricated Pt electrodes could be reassembled on the same MoS_2_ channel using vdW integration technique^35^ (Fig. 2d). The high work function of Pt (5.7 eV) electrodes matches well with the valance band of MoS_2_, hence the majority carrier type can be switched from electron to hole^35–37^. As shown in Fig. 2e, using the same MoS_2_ flake as the channel, the device is reconfigured into well-behaved p-type transistor with linear *I*_ds_–*V*_ds_ output curve, suggesting the p-type ohmic contact within vdW MoS_2_/Pt junctions (Fig. 2e, inset). Importantly, the threshold voltage of the reconfigured transistor is also close to 0, indicating the vdW disassembling and re-integrating processes could maintain the intrinsic properties of MoS_2_ channel. We note the vdW integration of different metal electrodes here is not only important to achieve atomically flat metal/MoS_2_ interfaces to avoid the Fermi level pinning effect and to achieve the polarity control, but also essential to achieve weakly bonded metal/MoS_2_ contact for successfully disassembling process. This is intrinsically different from conventional transistor with strongly bonded metal/semiconductor interfaces (typically achieved through high energy sputtering or vacuum deposition), where the electrodes can not be removed once deposited and the device function (or transistor polarity) is fixed once fabricated^35,38,39^.

Using this technique, the as-measured p-type transistor is disassembled again (Fig. 2f), and the pristine MoS_2_ channel could be further integrated with top-gate stacks, resulting in the tunable device geometry from back-gate device to dual-gate structure. As shown in Fig. 2g, the top-gate stack is pre-fabricated on sacrifice wafer, consisting top-gate dielectric (20 nm BN) and gate electrodes (Ag/Au, 30/20 nm). After the gate stack is laminated on the previous disassembled MoS_2_ channel (with pristine surface) (Fig. 2h), the dual-gate transistor electrical measurement is conducted immediately. Figure 2i shows n-type *I*_ds_–*V*_tg_ transfer curve of the device, where the top-gate strongly modulates the channel region. The inset shows 2D plot of the *I*_ds_ as a function of top-gate voltage (*V*_tg_) and back-gate voltage (*V*_bg_), from which we can determine the slope of threshold voltage shift. The slope gives the ratio between top-gate and back-gate capacitances, *C*_tg_∕*C*_bg_ ~17. Using the back-gate capacitance value of *C*_bg_ of 9.6 nF cm^−2^ (300 nm SiO_2_ and 60 nm BN), the top-gate capacitance is calculated to be 163.2 nF cm^−2^. This corresponds to a 21.7 nm thick BN, consistent with top-BN used here (20 nm identified by AFM and optical contrast), further indicating the well-behaved dual-gate device functions of reassembled vdWHs.

Moreover, we have also performed the AFM measurement of the MoS_2_ surface after disassembling both Ag electrodes and Pt electrodes on the same MoS_2_ channel, as shown in Supplementary Fig. 3. The MoS_2_ channel still demonstrates pristine surface after continuous assembling and disassembling both Ag and Pt electrodes, further confirming the mechanical assembling/disassembling approach without contaminations and interfaces residues.

### Reliability and stability of the disassembling technique

To confirm the high reliability of our assembling/disassembling processes, we have disassembled/reassembled a MoS_2_ transistor 10 times by repeatedly vdW integrating/releasing Ag electrode pairs on the same MoS_2_ channel. The optical images after every assembling/disassembling process are shown in Supplementary Fig. 4, and the channel region does not show observable damages after ten repetitive processes. Importantly, the electrical measurement is also directly conducted after each assembling process, where the reliability and stability of the assembling/disassembling approach could be evaluated.

The *I*_ds_–*V*_gs_ transfer curve and *I*_ds_–*V*_ds_ output curve of the MoS_2_ transistor after each integration process are shown in Supplementary Fig. 5a–j, from which the key device parameters could be extracted. The relationship of two-terminal mobility with different assembling times is demonstrated in Supplementary Fig. 5k, where the extracted values are relatively consistent around 50 cm^2^ V^−1^ s^−1^ for each measurement (in the range between 45 to 57 cm^2^ V^−1^ s^−1^). Similarly, other key device parameters (on-off ratio, on-state resistance, hysteresis) are also summarized in Supplementary Fig. 5l–n, and these figure-of-merit parameters also remain stable between different assembling processes, indicating relatively high reliability and stability of our assembling/disassembling processes. We note the finite cycle-to-cycle variations could originate from the variation of channel location during each integration process, since the alignment transfer techniques have a typical accuracy around 1 μm and the exactly same channel location can’t be ensured each time; or could be attributed to the variation of Ag electrodes quality (such as its bottom surface roughness or crystallinity).

### Reconfigurable electronic devices of a four-layer vdWH

**Fig. 3:**
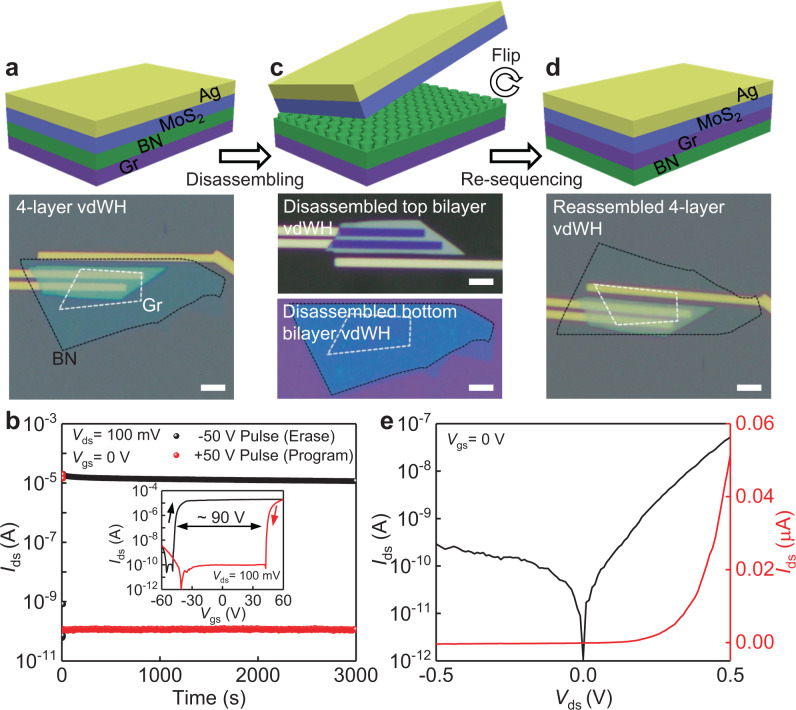
Reconfigurable electronic devices by disassembling and reassembling a four-layer vdWH. **a** Schematic and the optical image of a floating gate transistor using a four-layer vdWH of graphene/BN/MoS_2_/Ag, where the graphene (Gr), BN, MoS_2_, and Ag are used as floating gate, tunneling dielectric, semiconductor channel, and metal contact, respectively. **b** Retention performance of the floating gate transistor (initial four-layer vdWH) and the inset shows transfer characteristics of the floating gate transistor. **c** Schematic and the optical images of four-layer vdWH separated into two individual bilayer vdWHs (MoS_2_/Ag in top and graphene/BN in bottom). **d** Schematic and the optical image of a reassembled four-layer vdWH with a new sequence (BN/graphene/MoS_2_/Ag) using the same building blocks, representing a Schottky diode device function. **e** Output characteristics (semi-log and linear plots) of the reassembled four-layer vdWH diode. Scale bars are 5 μm in optical images.

Beyond reassembling and re-plugging into various combinations in bilayer vdWHs, this technique can be applied to multi-layer vdWHs by changing the stacking sequence using same building blocks. For example, we create a four-layer vdWH (graphene/BN/MoS_2_/Ag) memory device^40^ on PVA substrate using vdW integration techniques (detailed in Methods and Supplementary Fig. 6), where the graphene, BN, MoS_2_, and Ag are used as floating gate, tunneling dielectric, semiconductor channel, and metal contact, respectively (Fig. 3a). The device shows a large memory window of 90 V by using back control gate, as shown in Fig. 3b. Furthermore, the program and erase states show a remarkable program/erase (P/E) current ratio exceeding 10^5^, allowing easy readout of the device state. Importantly, the large P/E ratio is retained after over 3000 s measurement, indicating the high durability of the fabricated vdWH memory.

Furthermore, applying the demonstrated disassembling technique, we could separate the four-layer vdWH (with the sequence of graphene/BN/MoS_2_/Ag) into two individual bilayer vdWHs, where one composes graphene/BN and the other is MoS_2_/Ag, as shown in Fig. 3c. The separation of four-layer vdWHs is well-controlled to happen only within middle BN/MoS_2_ interface rather than in bottom graphene/BN or top MoS_2_/Ag interfaces, by carefully designing the interaction area (hence the vdW forces) of each vdW interface, as shown in Supplementary Fig. 6. Next, the disassembled building block graphene/BN is flipped upside down into BN/graphene and stacked with the disassembled MoS_2_/Ag again, therefore the stacking sequence is changed into BN/graphene/MoS_2_/Ag with essentially same 2D flakes, as shown in Fig. 3d. This new device structure can function as a Schottky diode, where the BN serves as substrate dielectric, graphene and MoS_2_ form PN diode and Ag is used as the electrical contact of MoS_2_. Due to the Fermi level difference between graphene and n-type MoS_2_, the device shows well-behaved n-type Schottky diode function with rectification ratio over 10^2^ (Fig. 3e), consistent with previous reports^41,42^. We note the demonstrated reconfigurable devices from memory to diode only represent two different sequences (out of 12 different sequences using a four-layer vdWH), and more device functions are expected if re-sequenced into different device structures, such as Ag/graphene/BN/MoS_2_ tunneling transistor^43^, or Ag/BN/MoS_2_/graphene photodetectors^44,45^. The choice of stacking sequences (hence device structures and functions) could increase exponentially with more layer numbers, offering another degree of freedom for constructing multi-functional vdWHs devices using same material building blocks.

### Reconfigurable anisotropic Raman behaviors

**Fig. 4:**
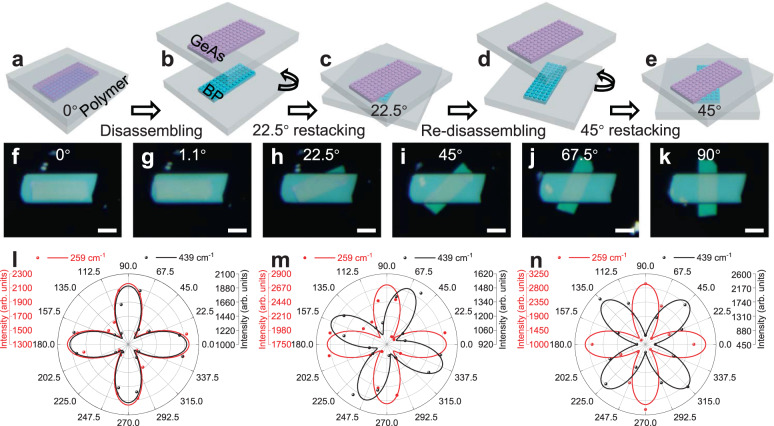
Disassembling and re-twisting of vdWHs. **a**–**e** Schematic illustration of the fabrication process of GeAs/BP vdWHs with different twist angles with five steps: the stacked GeAs/BP heterostructure (**a**); GeAs separated from BP (**b**); BP rotated 22.5° and re-stacked to form GeAs/BP heterostructure (**c**); GeAs separated from BP again(**d**); BP rotated 45° and re-stacked into GeAs/BP heterostructure (**e**). **f**–**k** Optical images of GeAs/BP vdWHs with various twist angles of 0° (**f**), 1.1° (**g**), 22.5° (**h**), 45° (**i**), 67.5° (**j**), and 90° (**k**). Scale bars are 5 μm. **l**–**n** Polar plots of Raman intensity of GeAs/BP heterostructures with various twist angles of 0° (**l**), 22.5° (**m**) and 45° (**n**).

Finally, besides re-combination and re-sequencing, the disassembled building blocks could be stacked again with different angles, leading to controllable twistable electronics with high twisting resolution. To demonstate this, two layered materials germanium arsenide (GeAs, ~25 nm thick) and black phosphorus (BP, ~10 nm thick) are used, and both materials demonstrate highly anisotropic lattice structures. The GeAs/BP vdWH is stacked using stardard dry-alignment transfer setup (see Methods). After disassembling the vdWH, the bottom BP and its holding substrate are rotated with any desired angle by a motor-controlled rotator with a resolution ~0.1° (Fig. 4a–e). As demonstrated in Fig. 4f–k and Supplementary Fig. 7, we have re-twisted the vdWH 16 times with a rotation spacing of 22.5°. We could measure the Raman spectrum of the vdWH under every twist angle, leading to the observation of switchable anisotropic behaviors within vdWHs. For example, when the GeAs and BP are alinged at 0 degree (alignment between the zigzag direction^46,47^), the vdWH demonstates a typical fourfold anisotropic Raman behavior, as shown in Fig. 4l. When the two materials are rotated to 22.5° and 45°, the symmetry of Raman signal is broken owing to the misalignment of two lattice structures, where the vdWHs exhibit eightfold anisotropic Raman signals, as shown in Fig. 4m and Fig. 4n, respectively. The polarized Raman spectra of GeAs/BP heterostructures with various twist angles are shown in Supplementary Fig. 8.

In particular, we have also demonstrated a rotation angle of 1.1° (Fig. 4g), which is reocongnized as magic angle in twisted bilayer graphene^21,22,48^. Although we do not observe novel physics in this stacking angle (between GeAs and BP), the highly controllable rotation technique could offer a new approch for investigating the rich physics within twistable electronics. Previous twistable electronics are largely achieved through two different approaches: one relies on fabricating and measuring large amounts of samples with different twist angles^24,49^, and the other relies on in situ mechanically pushing the etched 2D materials using AFM tip^27^. Although these approaches have successfully demonstrated interesting physics beyond the reach of existing materials, they are still limited by the complex fabrication and twisting process, as well as the highly variable material qualities (e.g., defects, dopants) and interface conditions (e.g., air bubble, surface contaminations) across different samples^50–52^. Within our simple approach, identical material quality is always ensured (because of the same material building blocks used) and the twisting angle is well-controlled. Based on this, the impact of small rotation angle change (e.g., change from 1.1° to 1.15°) to the lattice commensuration or moiré pattern generation would be an interesting topic for further investigation, which is difficult to achieve using previous techniques. We note the rotation resolution (currently 0.1°) could also be improved with better motor rotator, which could reach an ultra-high resolution below 0.01° in commercialized products.

## Discussion

In summary, we report a general technique for disassembling the artificial stacked vdWHs into individual building blocks, which can be further reassembled into new vdWHs via re-combining, re-sequencing, and re-twisting, hence new device functions. This method shows high reliability and stability, as verified by the multiple disassembling and reassembling processes and the corresponding AFM measurement. Within this, we have demonstrated reconfigurable transistors functions (n-type to p-type, back-gate to dual-gate) in a bilayer vdWH through re-stacking; reconfigurable device behaviors (floating gate memory to Schottky diode) in a four-layer vdWH through re-sequencing; and reconfigurable anisotropic Raman behaviors (fourfold to eightfold) through re-twisting, using same material building blocks. These proof-of-concept devices not only demonstrate the possibility to manipulate and re-stack diverse material layers at atomic level, but could also intrigue a research field of disassembled/reassembled vdWHs electronics in parallel of state-of-the-art vdWHs electronics, providing rich implications for multi-functional pluggable electronics, optoelectronics with limited material building blocks.

## Methods

### Disassembling process of a bilayer vdWH

First, thin PVA layer (~8 nm thick) is spin-coated on SiO_2_ substrate, where a MoS_2_ flake can be further mechanically exfoliated on top. The spin-coated PVA is atomically flat, with a root-mean-square surface roughness of 0.25 nm (Supplementary Fig. 9). It is noted that the PVA layer is essential here to fix the peeled MoS_2_, owing to its high adhesion forces. Next, a few-layer WSe_2_ or BN (~6 nm or 32 nm, respectively) is mechanically exfoliated on top of sacrifice SiO_2_ substrate and then functionalized by a hexamethyldisilazane (HMDS) layer in a sealed chamber at 80 °C for about 5 min. Next, the wafer is covered by a PVA layer (~2 μm thick) through spin-coating, where the PVA layer (used as the handling substrate) with WSe_2_ or BN wrapped underneath is mechanically released and transferred on top of MoS_2_. Once the vdWH formed, the PVA layer can be further removed (together with WSe_2_ or BN wrapped underneath) using thermal release tape, representing the disassembling process of the vdWH.

### Fabrication process of a reconfigurable MoS_2_ device with different contact metals

First, a few-layer MoS_2_ is exfoliated on 300 nm SiO_2_/p^++^ Si substrate (covered with a thin layer of BN). Next, metal electrodes (50 nm thick Pt or 30/20 nm thick Ag/Au) are pre-fabricated on sacrificial substrate through electron-beam evaporation, which can be mechanically released from the substrate and physically laminated on MoS_2_, resulting in an atomically sharp and clean metal-2D material interface. After that, the poly(methyl methacrylate) (PMMA) on top of the metal pads is removed using electron-beam lithography and development processes for electrical probing and measurements.

To disassembled the vdWH, the transferred metal electrodes are mechanically separated from the MoS_2_ channel using a thermal release tape. We note that the PMMA on top of BN is removed using standard electron-beam lithography and development processes before the disassembling process to prevent the channel material being taken away. Pre-fabricated Pt electrodes and top-gate stacks are laminated and disassembled using the same method described above.

### Disassembling and reassembling processes of a four-layer vdWH

First, monolayer graphene is mechanically exfoliated onto PVA (~8 nm)/SiO_2_ (300 nm) substrate, and then 13 nm thick BN is dry transferred onto as-exfoliated graphene, leading to the formation of graphene/BN vdWH. Next, MoS_2_/Ag vdWH is pre-fabricated on top of a sacrificial substrate, which can be further mechanically released and laminated onto the graphene/BN vdWH, resulting in a four-layer (graphene/BN/MoS_2_/Ag) vdW non-volatile memory.

To disassemble the four-layer vdWH, the upper two layers (MoS_2_/Ag) are mechanically separated from BN by using thermal release tape to pick up PMMA layer. The PMMA layer (together with the MoS_2_/Ag vdWH) is further transferred onto a SiO_2_ substrate by removing thermal release tape under 100 °C. Next, 500 nm thick PMMA is spin-coated on the bottom vdWH (graphene/BN), and the substrate is immersed into an 80 °C deionized water bath for 30 min to dissolve the PVA underneath. Once PVA is dissolved, the PMMA membrane will float on top of water, which can be further flipped and fished using a Si wafer (with 100 nm thick SiO_2_). After that, the substrate is heated to 120 °C for 10 min to fully remove any water adsorbed on BN/graphene vdHW. Finally, the pre-disassembled MoS_2_/Ag is re-stacked onto the BN/graphene, resulting in a four-layer (BN/graphene/MoS_2_/Ag) vdW diode. The corresponding schematics and optical images are shown in Supplementary Fig. 6.

### Disassembling and re-twisting of vdWH

Multi-layer GeAs and BP are mechanically exfoliated on two sacrifice wafers, respectively. Next, the wafers are functionalized by HMDS and spin-coated with a PMMA layer, where the PMMA layer with BP wrapped underneath is picked up using transparent double-sided tape, and the tape with the BP side up is fixed on a slide glass. Afterward, the PMMA layer with GeAs wrapped underneath is mechanically released and transferred onto BP. Subsequently, the PMMA layer with GeAs can be mechanically separated from BP using Scotch tape, placed on PDMS and transferred onto BP again under optical microscope. GeAs/BP vdWHs with various twist angles can be achieved by repeating the above approach multiple times, as shown in Supplementary Fig. 7. Besides, PVA could also be used as the handling substrate for re-twisting electronics. As shown in Supplementary Fig. 10, we have used PVA substrate to fabricate twisted GeAs/BP vdWHs with same fabrication process, except PMMA is changed into PVA.

### Materials characterizations and electrical measurements

An atomic force microscope (Bioscope system, Brucker) is used to characterize the morphology and thickness. Raman spectrum studies of GeAs/BP vdWHs are conducted using a confocal microscope (Renishaw invia-reflex) excited by 633 nm laser and angle dependent polarized Raman spectra are obtained by precisely rotating sample orientation. Electrical measurements are carried out in probe station (Lakeshore, PS-100) at room temperature, using Keysight B2912A source measurement unit and Agilent B1500A semiconductor analyzer.

## Supplementary information

Supplementary Information

## Data Availability

The data that support the findings of this study are available from the corresponding author upon reasonable request.
