# Supplementary material for: Reconfigurable electronics by disassembling and reassembling van der Waals heterostructures
Source: Nat Commun. 2021 Mar 23;12:1825. doi: 10.1038/s41467-021-22118-y (PMC7988143; doi:10.1038/s41467-021-22118-y)
Supplement: Supplementary file 1 — Supplementary Information [file 41467_2021_22118_MOESM1_ESM.pdf]

Supplementary information for

Reconfigurable electronics by disassembling and reassembling van der Waals heterostructures

Quanyang Tao, Ruixia Wu, Qianyuan Li, Lingan Kong, Yang Chen, Jiayang Jiang, Zheyi Lu, Bailing Li, Wanying Li, Zhiwei Li, Liting Liu, Xidong Duan, Lei Liao & Yuan Liu*

*Corresponding author. E-mail: yuanliuhnu@hnu.edu.cn

Contents:

Supplementary Figures 1–10

Supplementary Figures

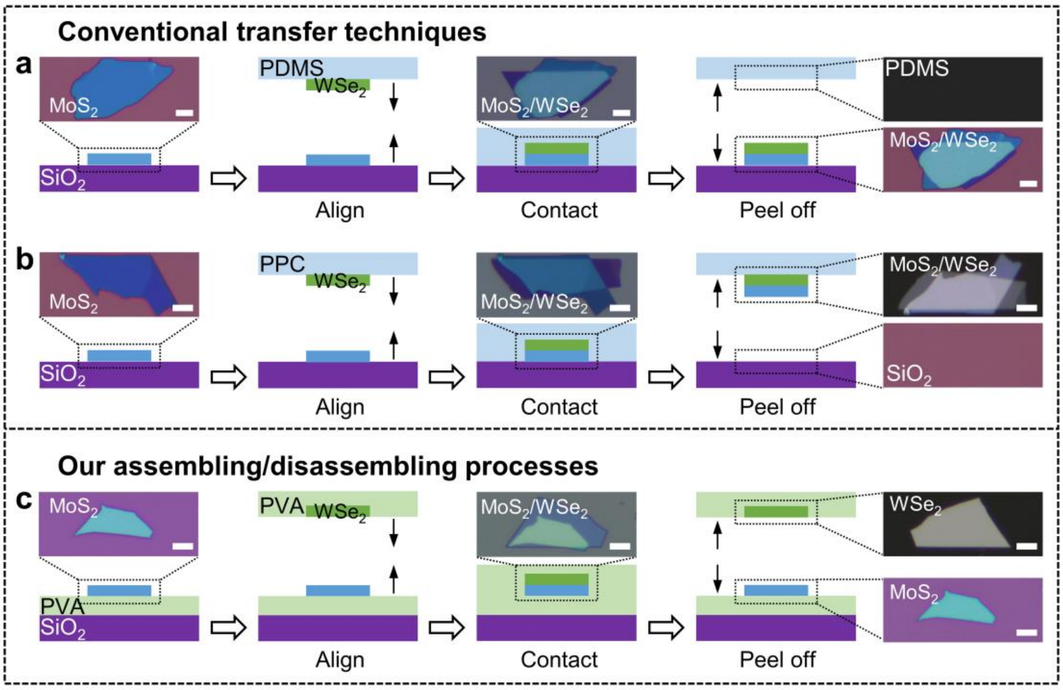

Supplementary Figure 1. Illustration of conventional transfer techniques and our assembling/disassembling processes. **a**, Conventional transfer process of a MoS_2/WSe_2 vdWH using PDMS as the handling polymer. With weaker PDMS/WSe_2 interaction force (compared to stronger MoS_2/WSe_2 vdW force), the whole vdWH would be released from the PDMS substrate when peeling off PDMS. Therefore, the vdWH can not be disassembled or further re-assembled. **b**, Conventional transfer process of a MoS_2/WSe_2 vdWH using PPC as the handling polymer. With weaker SiO_2/MoS_2 interaction force (compared to stronger MoS_2/WSe_2 vdW force), the whole vdWH would be picked up by PPC polymer when peeling off. Therefore, the vdWH can not be disassembled or further re-assembled. **c**, Our assembling/disassembling processes using PVA as both the substrate and handling polymer. With stronger PVA/2D interaction force (compared to MoS_2/WSe_2 vdW force), the as assembled vdWH would be separated when peeling off the handling substrate, resulting in the successfully disassembling of vdWH and coule be further used as individual blocks for vdW integration. Scale bars are 5 μm in optical images.

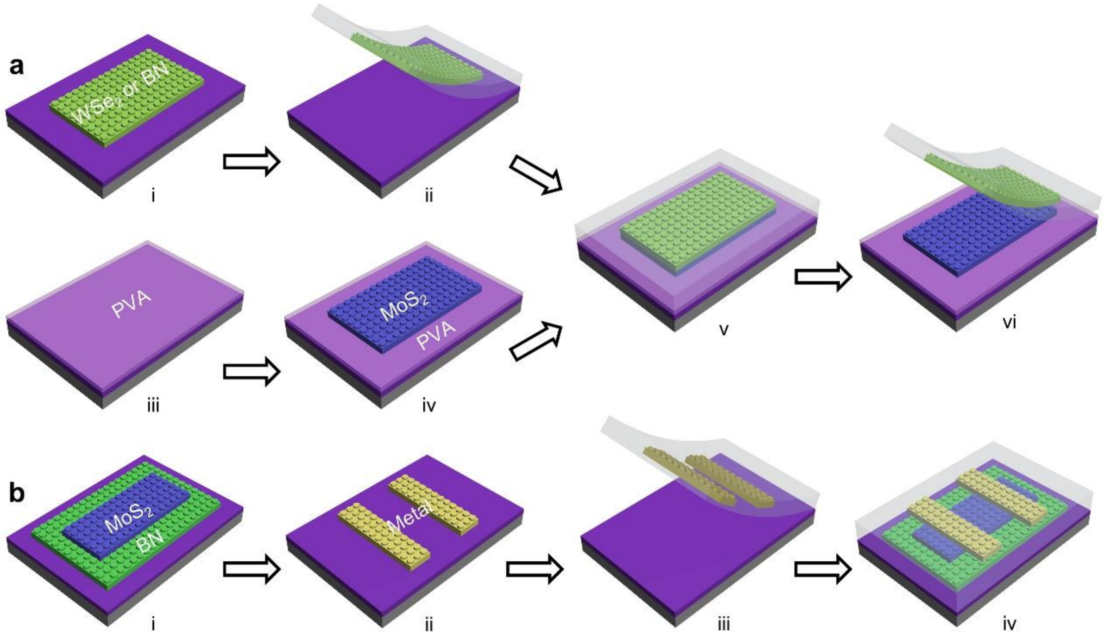

Supplementary Figure 2. Assembling and disassembling processes. a, Schematic illustration of disassembling process of bi-layer vdWH: (i) WSe_2 or BN exfoliation on SiO_2 substrate; (ii) peeling off WSe_2 or BN with the assisted of PVA; (iii) PVA spin-coated on SiO_2 substrate; (iv) MoS_2 exfoliation on PVA/ SiO_2 substrate; (v) WSe_2 or BN transferred onto MoS_2, (vi) WSe_2 or BN disassembled from the bottom MoS_2. **b**, Schematic illustration of metal–semiconductor junctions integration process: (i) MoS_2/BN vdWH on SiO_2 substrate; (ii) metal deposition on sacrificial substrate, (iii) peeling off the metal with the assisted of PMMA; (iv) Metal transferred onto the MoS_2/BN vdWH.

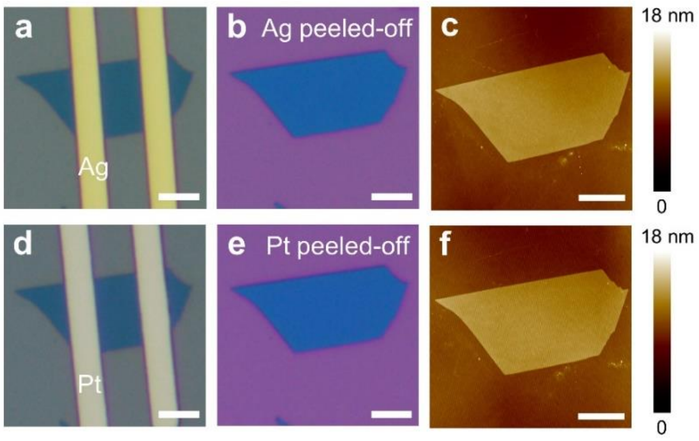

Supplementary Figure 3. Morphological characterizations of vdW metal–semiconductor junctions after continuous assembling/disassembling processes. a, b, Optical images of a MoS_2 flake assembled with Ag electrodes (**a**) and then disassembled (**b**). **c,** AFM topography image of the disassembled MoS_2 flake, demonstrating clean interfaces. **d, e,** Optical images of the same MoS_2 flake after assembled with Pt electrodes (**d**) and then disassembled (**e**). **f,** AFM topography image of the disassembled MoS_2 flake again, where clean surface is observed. Scale bars are 5 μm in **a**–**f**.

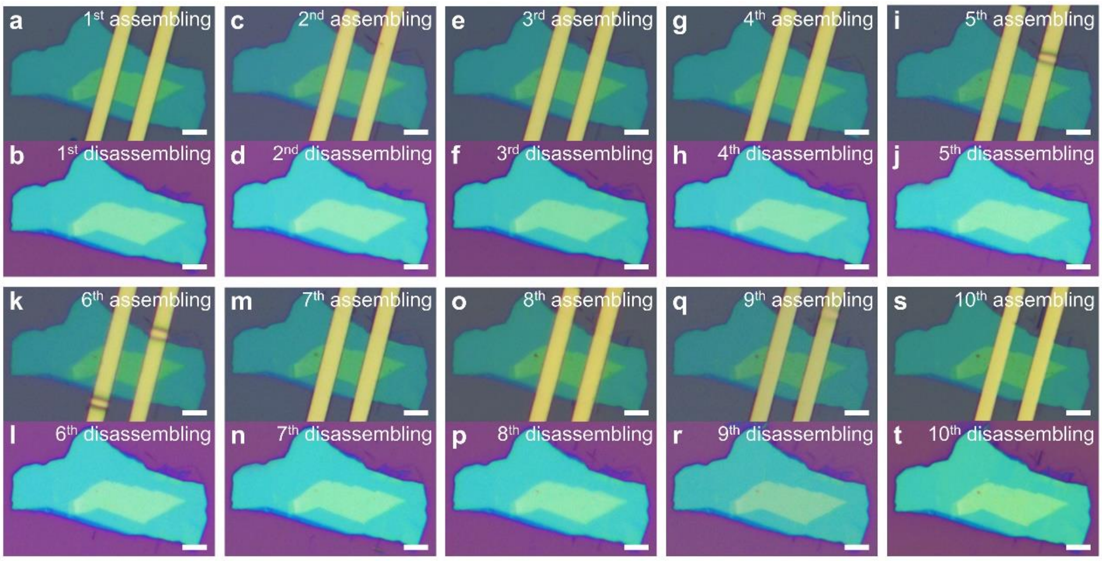

Supplementary Figure 4. Repetitive assembling/disassembling processes. Optical images of a MoS_2 transistor after repetitive assembling/disassembling processes, where **a** and **b** are images after 1st process, **c** and **d** are images after 2nd process, **e** and **f** are images after 3rd process , **g** and **h** are images after 4th process, **i** and **j** are images after 5th process, **k** and **l** are images after 6th process, **m** and **n** are images after 7th process, **o** and **p** are images after 8th process, **q** and **r** are images after 9th process, **s** and **t** are images after 10th process. Scale bars are 5 μm.

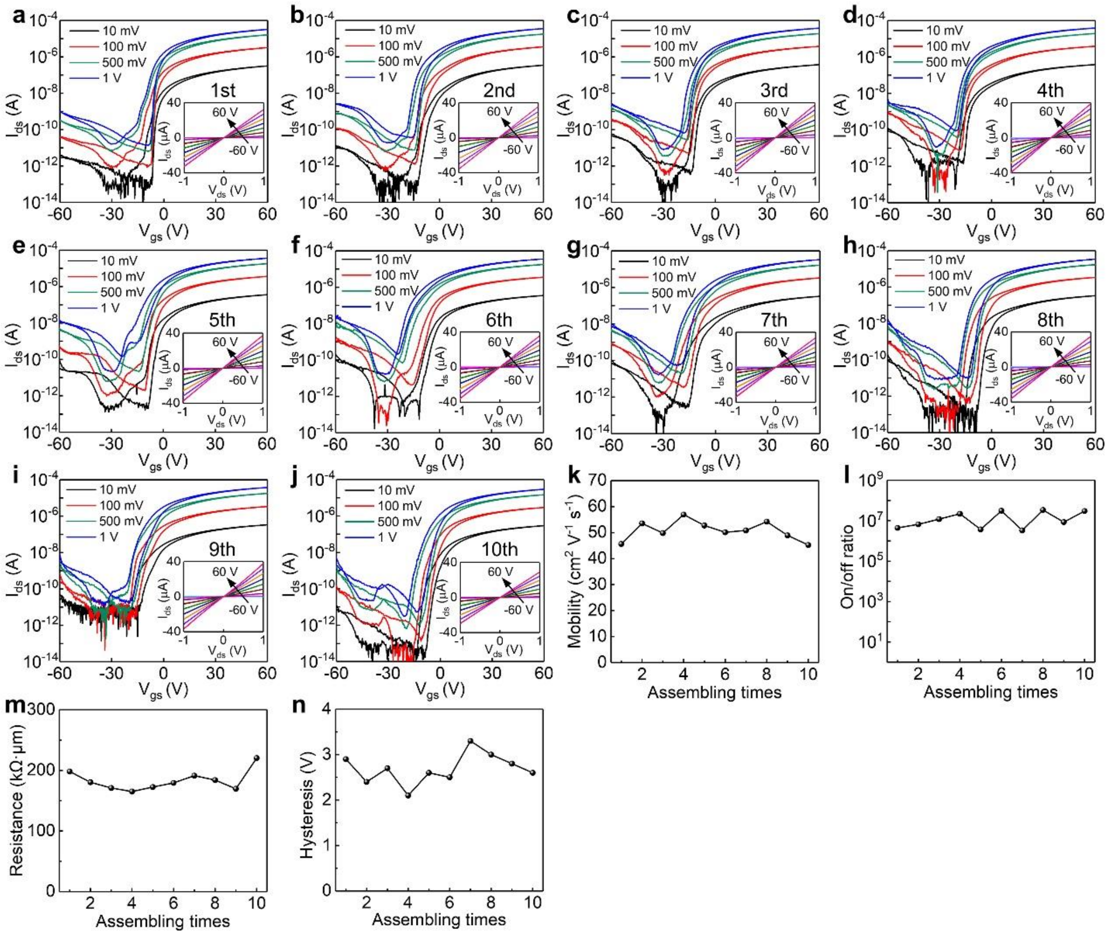

Supplementary Figure 5. The electrical reliability and stability measurement of a MoS_2 transistor after 10 assembling/dis-assembling processes using the same MoS_2 channel. a–j, I_{ds}–V_{gs} transfer curve and I_{ds}–V_{ds} output curve (inset) of the MoS_2 transistor after each integration process. For I_{ds}–V_{ds} output curves, various gate voltages from -60 V to 60 V are applied with a step of 10 V. **k–n,** The relationship of two-terminal mobility (**k**), on-off ratio (**l**), on-state resistance (**m**) and hysteresis (**n**) with assembling times. The on-state resistance is extracted under 60 V gate and 1 V source drain bias. The gate voltage hysteresis is extracted under 0.1 V source drain bias and 1 μA source drain current.

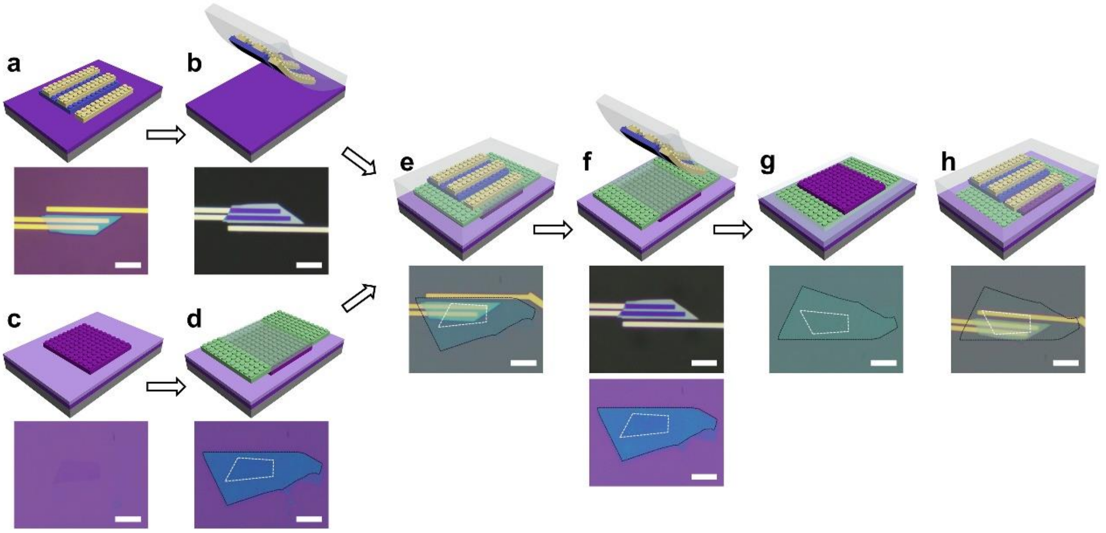

Supplementary Figure 6. Schematic illustration and optical images of disassembling and reassembling of four-layer vdWH. a, **b**, Ag electrodes are deposited on a few layer MoS_2 flake to form MoS_2/Ag vdWH (**a**), and then mechanically peeled off with the assisted of the PMMA (**b**). **c**, **d**, Another monolayer graphene is exfoliated on PVA-coated SiO_2 (**c**), where a BN flake can be further transferred on top to form a graphene/BN vdWH (**d**). **e**, The MoS_2/Ag vdWH is dry transferred onto graphene/BN vdWH, leading to the formation of a four-layer vdWH (graphene/BN/MoS_2/Ag) and a floating gate memory. **f**, The MoS_2/Ag vdWH can be further separated from graphene/BN heterostructure. The separation is controlled to happen between BN and MoS_2 interfaces owing to two reasons. First, the PVA/BN has larger integration area compared to MoS_2/BN, hence stronger adhesion force. Second, the PMMA/Ag has larger integration area compared to PVA/Ag and Ag is strongly bonded to MoS_2, therefore larger PMMA/MoS_2 adhesion force is expected. **g**, The disassembled graphene/BN vdWH (together with PMMA membrane) is flipped upside down (into BN/graphene vdWH) and transferred onto SiO_2 substrate. **h**, The disassembled MoS_2/Ag vdWH is reassembled again on the BN/graphene vdWH, leading to the formation of BN/graphene/MoS_2/Ag vdW diodes. Scale bars are 10 μm in optical images.

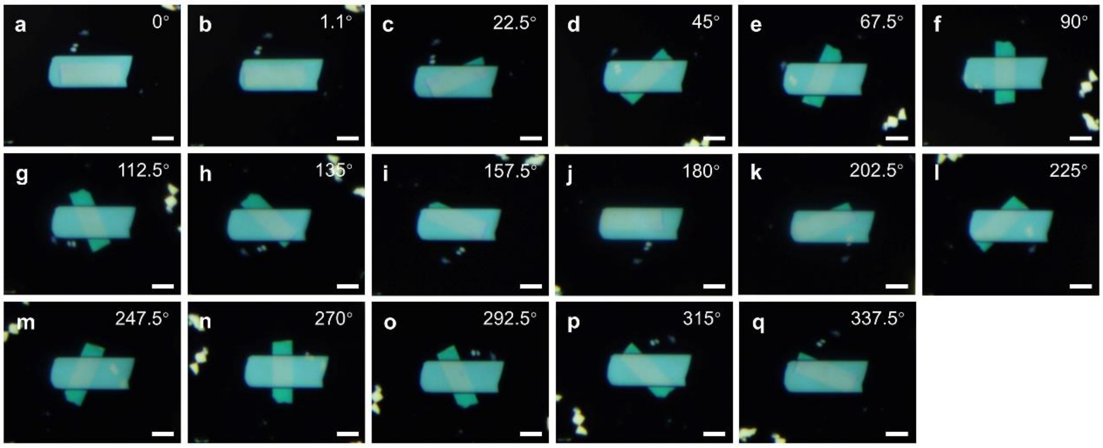

Supplementary Figure 7. GeAs/BP vdWHs with various twist angles. a–q, Optical images of GeAs/BP **vdWHs with various twist angles** from 0° to 337.5° with a twisting step of 22.5°. The GeAs is on top and BP is on bottom. Scale bars are 5 μm.

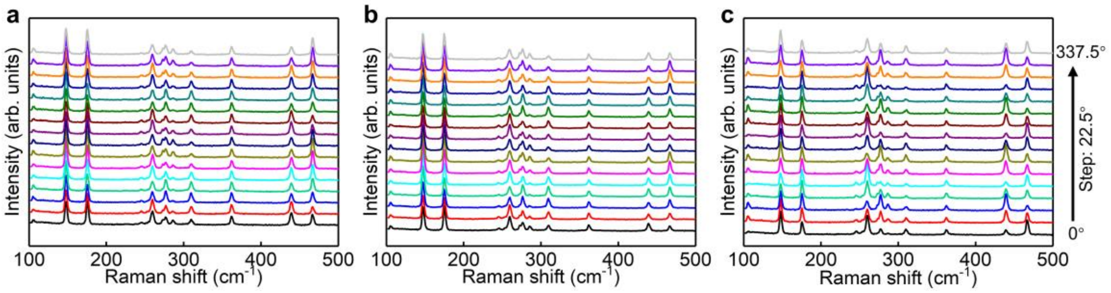

Supplementary Figure 8. Raman spectra of GeAs/BP heterostructures. Polarized Raman spectra of GeAs/BP heterostructures with different twist angles of 0° (**a**), 22.5° (**b**) and 45° (**c**), with typical Raman peaks at 106, 148, 176, 259, 273, 277, 286 and 310 cm^{-1} for GeAs, and typical Raman peaks at 362, 439 and 467 cm^{-1} for BP.

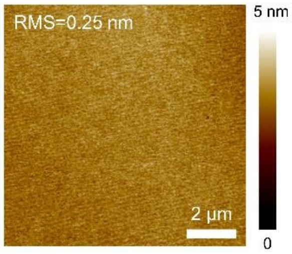

Supplementary Figure 9. AFM topography image of the spin-coated PVA film, with a surface roughness of 0.25 nm.

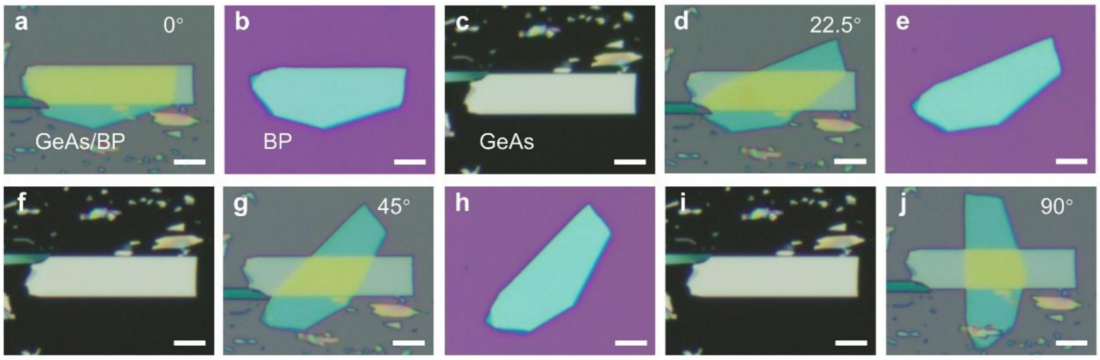

Supplementary Figure 10. Disassembling and re-twisting GeAs/BP vdWHs using PVA substrate. a, Optical image of the stacked GeAs/BP heterostructure at 0°. **b, c,** Optical images of the disassembled BP on PVA substrate (**b**) and disassembled GeAs (**c**). **d,** Optical image of the re-stacked GeAs/BP heterostructure at 22.5°. **e, f,** Optical images of the disassembled BP (**e**) and GeAs (**f**) flake. **g,** Optical image of the re-stacked GeAs/BP heterostructure at 45°. **h, i,** Optical images of the disassembled BP (**h**) and GeAs (**i**) flake. **j,** Optical image of the re-stacked GeAs/BP heterostructure at 90°.
